# Long-term outcomes of very low birth weight infants with intraventricular hemorrhage: a nationwide population study from 2011 to 2019

**DOI:** 10.1007/s12519-024-00799-x

**Published:** 2024-04-13

**Authors:** Joonsik Park, Sook-Hyun Park, Yu-ra Kwon, So Jin Yoon, Joo Hee Lim, Jung Ho Han, Jeong Eun Shin, Ho Seon Eun, Min Soo Park, Soon Min Lee

**Affiliations:** Department of Pediatrics, Yonsei University College of Medicine, 211 Eonjuro Gangnamgu, Seoul, 06273 Republic of Korea

**Keywords:** Cerebral intraventricular hemorrhage, Developmental, Hydrocephalus, Mortality, Posthemorrhagic ventricular dilatation, Surgery

## Abstract

**Background:**

Advancements in neonatal care have increased preterm infant survival but paradoxically raised intraventricular hemorrhage (IVH) rates. This study explores IVH prevalence and long-term outcomes of very low birth weight (VLBW) infants in Korea over a decade.

**Methods:**

Using Korean National Health Insurance data (NHIS, 2010–2019), we identified 3372 VLBW infants with IVH among 4,129,808 live births. Health-related claims data, encompassing diagnostic codes, diagnostic test costs, and administered procedures were sourced from the NHIS database. The results of the developmental assessments  are categorized into four groups based on standard deviation (SD) scores. Neonatal characteristics and complications were compared among the groups. Logistic regression models were employed to identify significant changes in the incidence of complications and to calculate odds ratios with corresponding 95% confidence intervals for each risk factor associated with mortality and morbidity in IVH. Long-term growth and development were compared between the two groups (years 2010–2013 and 2014–2017).

**Results:**

IVH prevalence was 12% in VLBW and 16% in extremely low birth weight (ELBW) infants. Over the past decade, IVH rates increased significantly in ELBW infants (*P* = 0.0113), while mortality decreased (*P* = 0.0225). Major improvements in certain neurodevelopmental outcomes and reductions in early morbidities have been observed among VLBW infants with IVH. Ten percent of the population received surgical treatments such as external ventricular drainage (EVD) or a ventriculoperitoneal (VP) shunt, with the choice of treatment methods remaining consistent over time. The IVH with surgical intervention group exhibited higher incidences of delayed development, cerebral palsy, seizure disorder, and growth failure (height, weight, and head circumference) up to 72 months of age (*P* < 0.0001). Surgical treatments were also significantly associated with abnormal developmental screening test results.

**Conclusions:**

The neurodevelopmental outcomes of infants with IVH, especially those subjected to surgical treatments, continue to be a matter of concern. It is imperative to prioritize specialized care for patients receiving surgical treatments and closely monitor their growth and development after discharge to improve developmental prognosis.

Supplementary file2 (MP4 77987 kb)

**Supplementary Information:**

The online version contains supplementary material available at 10.1007/s12519-024-00799-x.

## Introduction

Intraventricular hemorrhage (IVH) in preterm infants occurs when a germinal matrix hemorrhage ruptures into the lateral ventricle through the ependyma [1]. Grades 3 and 4 (G3 and G4) IVH increase the risk of long-term neurologic and neurodevelopmental disability, including seizures, cognitive and executive function impairment, and cerebral palsy (CP) [2–4]. Advancements in neonatal intensive care, which have improved the survival rates of extremely preterm infants, have also resulted in a relative increase in the number of infants at high risk of developing IVH [5, 6]. The reported incidence of IVH in very low birth weight (VLBW) infants decreased from 50% in the early 1980s to around 20% in 2005 [7, 8]. However, more recent studies up to 2012 have reported a stable or increasing trend [3, 9]. According to data from the Korean Neonatal Network, the overall survival rate of the enrolled VLBW infants was 87% between 2013 and 2020. Although not statistically significant, the overall survival rate of preterm infants born in 2020 improved compared with that in the previous era [10]. The prevalence of overall IVH and IVH [≥ grade 2 (G2)] among VLBW infants was 42.5% and 17%, respectively. Among infants with a gestational age of less than 24 weeks, the prevalence of IVH (≥ G2) was 52% [11].

Posthemorrhagic ventricular dilatation (PHVD) is characterized by progressive ventricular enlargement and elevated intracranial pressure following hemorrhagic events. The incidence of PHVD with surgical intervention has remained stable nationwide and is reported to be 25%–50% in patients with IVH [12]. Although there is controversy regarding the impact and timing of neurosurgical interventions, direct removal or diversion of cerebrospinal fluid remains the primary approach to PHVD therapy [13]. Infants with severe IVH complicated by PHVD (40%–60%) are at significantly higher risk of poor neurodevelopmental outcomes, especially those who ultimately require a shunt (75%–88%) [14, 15]. Significant cognitive and motor impairments were found among infants with PHVD at 18–24 months’ corrected age [16–19]. However, there are limited nationwide studies on PHVD.

A deeper understanding of the changing epidemiology of IVH and PHVD among VLBW infants may enhance the measurement of disease burden, allocation of public health resources, and development of clinical research objectives. Therefore, we undertook this study to determine the prevalence of IVH in VLBW infants and investigate the long-term neurodevelopmental outcomes associated with severe IVH over a 10-year period in Korea.

## Methods

### Study population and design

We utilized the National Health Insurance Service (NHIS) database to identify VLBW infants diagnosed with IVH (≥ G2) [International Classification of Disease (ICD)-10 codes: P52.1, P52.2, P52.4, P52.5, P52.8, P52.9, I61.5] between 2010 and 2019. Health-related claims data, encompassing diagnostic codes, diagnostic test costs, and administered procedures were sourced from the NHIS database, which covers nearly all Korean residents and is linked to the National Health Screening Program for Infants and Children database. Information regarding birth weight was collected through ICD-10 codes provided by hospitals or via questionnaires administered by the Infant Health Screening Program. We excluded preterm infants with IVH of grade 1 (G1) severity, subependymal hemorrhage, IVH unspecified, or germinal matrix hemorrhage [ICD-10 codes: P52.0 (*n* = 6955) and P52.3 (*n* = 4002)]. Birth certificate data from Statistics Korea were utilized to estimate the prevalence of IVH (https://kosis.kr/statisticsList).

The National Health Screening Program for Infants and Children in Korea was initiated in 2007 as a primary clinical service aimed at monitoring current health issues [20]. This comprehensive program encompasses medical history interviews, physical examinations, anthropometric measurements, visual acuity screenings, developmental assessments using the Korean Developmental Screening Test (K-DST), oral examinations, and anticipatory guidance questionnaires [21]. The study population underwent their initial assessments at 4–6 months of age, followed by subsequent check-ups at 9–12 months, 18–24 months, 30–36 months, 42–48 months, 54–60 months, and 72 months. The K-DST is a screening tool designed to assess the normal neurodevelopmental status of infants across six domains: gross/fine motor skills, cognition, communication, social interaction, and self-control. The K-DST is administered according to the child's corrected age at the time of the clinic visit [22].

### Definition

The results of the K-DST are categorized into four groups based on standard deviation (SD) scores. Scores below − 2 SD warrant “further work-up”, while scores between − 2 and − 1 SD recommend “close observation”. Scores between − 1 and 1 SD indicate “peer-level” performance and scores above 1 SD are classified as “high-level”.

In this study, treatment strategies and the identification of long-term complications associated with IVH relied on hospital-recorded ICD-10 codes. These codes included: external ventricular drainage (EVD) (N0324), ventriculoperitoneal (VP) shunt (Z982, S4712), hyaline membrane disease (HMD) (P22.0), bronchopulmonary dysplasia (BPD) (P27.1), patent ductus arteriosus (PDA) ligation (O1671), sepsis (P36), necrotizing enterocolitis (NEC) (P77), periventricular leukomalacia (PVL) (P91.2), retinopathy of prematurity (ROP) (P35.1), delayed development (DD) (R62.9), cerebral palsy (CP) (G80), autism spectrum disorders (F84.9), sensorineural hearing loss (H90.5), blindness (H54.0), and seizure disorder (G40 or R56.8).

### Statistical analyses

We expressed the baseline characteristics of the patients as means and SDs for continuous variables and as percentages for categorical variables. The cohort was stratified based on birth weight or year. We used the chi-square test to compare neonatal characteristics and complications between the groups. Logistic regression models were employed to identify significant changes in the incidence of complications and to calculate odds ratios with corresponding 95% confidence intervals for each risk factor associated with mortality and morbidity in IVH.

To assess linear trends over time, we utilized the Cochran–Armitage trend test to determine whether each dependent variable exhibited significant changes over time. We also compared co-morbidities by dividing the 10-year period into two halves of 5 years each and conducted post-hoc analyses to determine differences between these two groups.

To facilitate the analysis of long-term morbidities, we excluded the most recent 2 years (2018 and 2019) from our dataset. This decision allowed us to observe the complete 3-year developmental trajectory of individual patients and capture any morbidities diagnosed later in life. For this analysis, we divided the data into two additional groups: the years 2010–2013 and 2014–2017, which enabled us to evaluate long-term growth and development.

All statistical analyses were carried out using SAS version 9.4 (SAS Institute, Cary, North Carolina). We considered *P*-values < 0.05 to be statistically significant.

### Ethics statement

We utilized National Health Insurance Service (NHIS) data (NHIS-2022–1-214) provided by the NHIS. The authors declare no conflicts of interest with the NHIS. To protect patient privacy, all identifiable variables, including claim-, individual-, and organizational-level identification numbers, were randomly regenerated by the NHIS database. The study protocol received approval from the Institutional Review Board (IRB) of Gangnam Severance Hospital (IRB No. 3–2021-0251). Given the retrospective study design, the requirement for informed consent was waived.

## Results

**Fig. 1:**
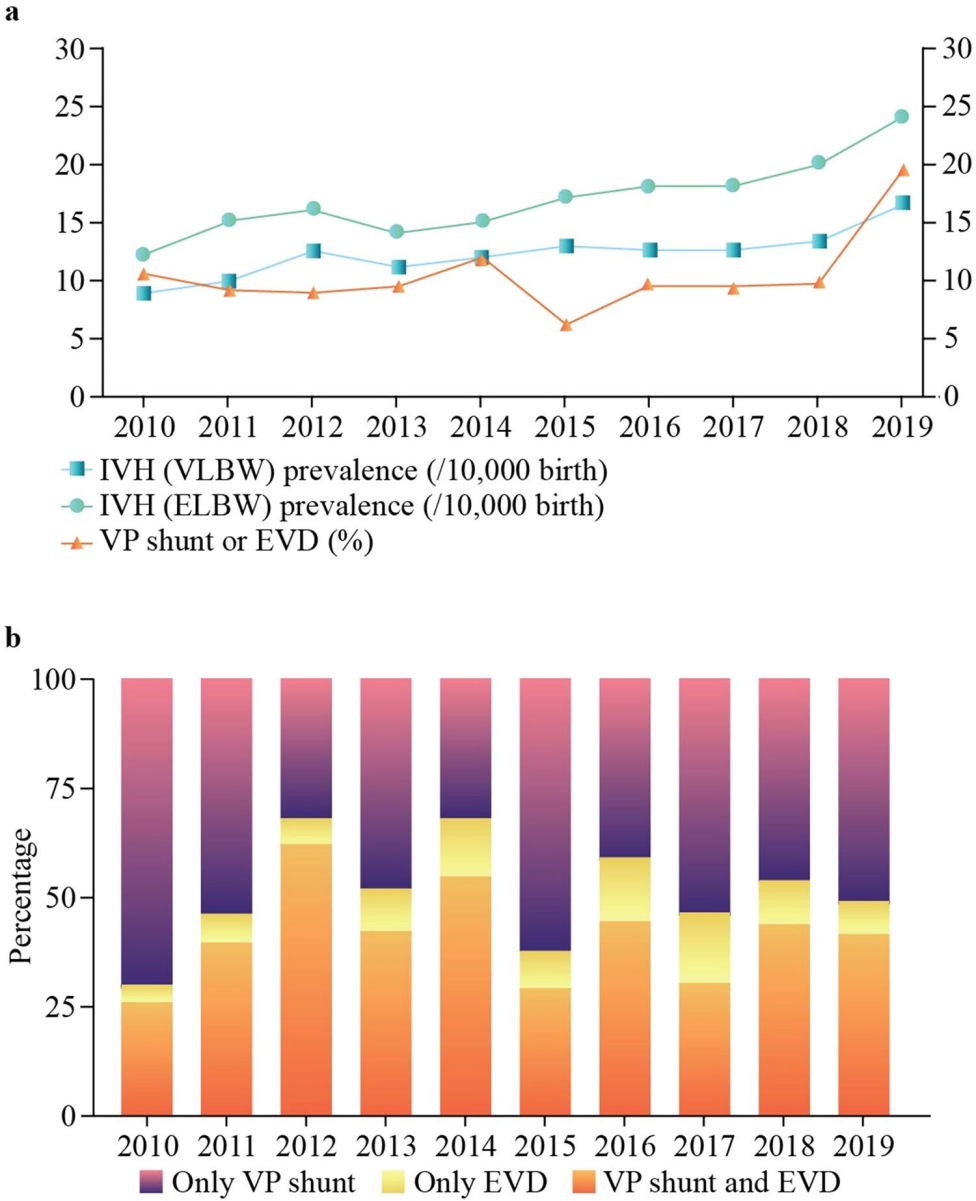
Trends of intraventricular hemorrhage and surgical treatment. *IVH* intraventricular hemorrhage, *VLBW* very low birth weight, *ELBW* extremely low birth weight, *VP* ventriculoperitoneal, *EVD* external ventricular drainage

Over the past 10 years in Korea, there were 3805 VLBW patients with G2–4 IVH and 2038 ELBW infants. The prevalence rate gradually increased from 2010 to 2019, as illustrated in Fig. 1. Among these patients, 321 (10.5%) received treatment with a VP shunt or EVD. Specifically, 154 patients (4.7%) received only a VP shunt, 31 patients (0.9%) underwent only EVD, and 136 patients (4.0%) were treated with both a VP shunt and EVD. It is worth noting that a significant increase in VP shunt placements was observed in the year 2019.

The co-morbidities observed in these patients included HMD in approximately 3320 (87%), PDA in 796 (21%), NEC in 121 (3%), and PVL in 561 (15%). BPD and ROP exhibited significant decreases according to the results of the linear trend test. When dividing the 10-year period into two halves of 5 years each, the results indicated a significant decrease in BPD from 79 to 57%, sepsis from 47 to 34%, and ROP from 72 to 57% in the later time period. Additionally, an analysis of the long-term prognosis for children born between 2010 and 2017 revealed a declining trend in the prevalence of CP, autism, and seizure disorder over time (Table 1).

**Table 1:** Co-morbidities and long-term outcomes among very low birth weight infants with intraventricular hemorrhage

Co-morbidities	Total (*n* = 3805) (2010–2019)	*P*-value^a^	2010–2014 (*n* = 1826)	2015–2019 (*n* = 1979)	*P*-value^b^
HMD	3320 (87%) (84%-81%)	0.470	1603 (88%)	1717 (87%)	0.343
BPD	2490 (65%) (79%-57%)	< 0.001	1274 (70%)	1216 (61%)	< 0.001
PDA ligation	796 (21%) (24%-21%)	0.995	379 (21%)	417 (21%)	0.811
Sepsis	1598 (42%) (47%-34%)	< 0.001	848 (46%)	758 (38%)	< 0.001
NEC	121(3%) (18%-12%)	0.320	304 (17%)	317 (16%)	0.599
PVL	561(15%) (12%-12%)	0.981	284 (16%)	292 (15%)	0.493
ROP	2243 (59%) (72%-57%)	0.001	1109 (61%)	1119 (57%)	0.009

Long-term outcomes	Total (*n* = 3019) (2010–2017)	*P*-value^a^	2010–2013 (*n* = 1429)	2015–2017 (*n* = 1590)	*P*-value^b^
Delayed development	1095 (36%) (33%-59%)	0.257	508 (36%)	587 (37%)	0.435
Cerebral palsy	837 (28%) (27%-43%)	< 0.001	433 (30%)	404 (25%)	0.003
Autism spectrum disorders	145 (5%) (3%-10%)	< 0.001	89 (6%)	56 (4%)	0.001
Sensoryneural hearing loss	306 (10%) (12%-18%)	0.153	135 (9%)	171 (11%)	0.235
Blindness	26 (1%) (0%- 2%)	0.049	17 (1%)	9 (0.6%)	0.064
Seizure disorder	926 (31%) (29%-48%)	< 0.001	479 (34%)	447 (28%)	< 0.001

Co-morbidities by dividing the 10-year period into two halves of 5 years each and long-term outcomes by dividing the 8-year period into two halves of 4 years each to observe the complete three-year developmental trajectory of individual patients

*HMD* hyaline membrane disease, *BPD* bronchopulmonary dysplasia, *PDA* patent ductus arteriosus, *NEC* necrotizing enterocolitis, *PVL* periventricular leukomalacia, *ROP* retinopathy of prematurity

^a^Cochran-Armitage trend test to examine linear trends over time

^b^Chi-square test

Among infants with IVH, an average of 92 patients (24%) were diagnosed with disabilities each year. The most prevalent disability diagnosis was cerebral lesion disorder, with an average of 66 patients (17%) registered annually, followed by intellectual disability in 15 patients (3%) and language disorder in eight patients (2%).

**Fig. 2:**
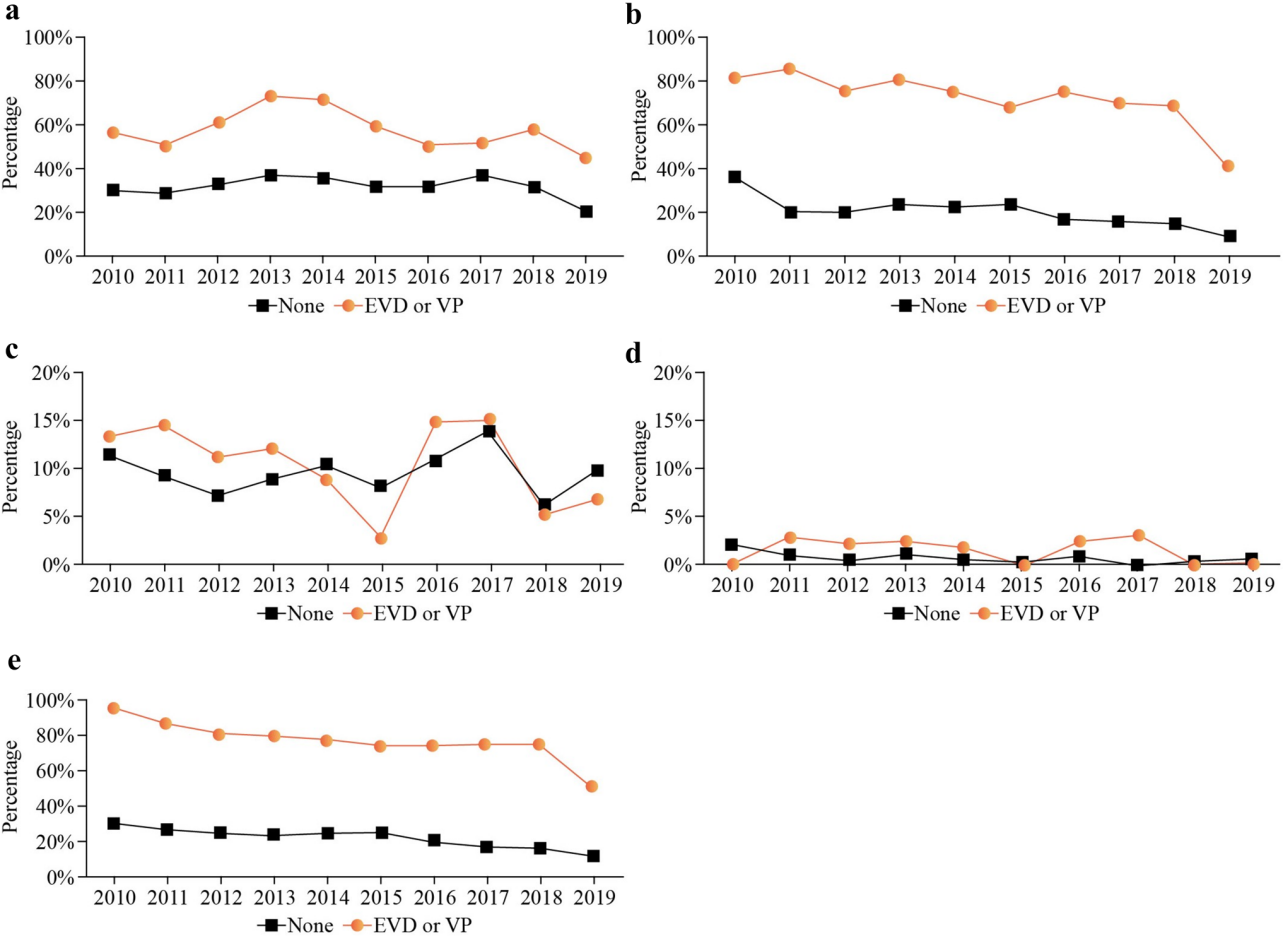
Trends of long-term neurological outcomes between infants with and without surgical treatment. **a** Delayed Development; **b** cerebral palsy; **c** sensorineural hearing loss; **d** blindness; **e** seizure disorder. *VP* ventriculoperitoneal, *EVD* external ventricular drainage

Over the 10-year period, it was confirmed that the prevalence of DD (*P* = 0.023), CP (*P* < 0.0001), epilepsy (*P* < 0.001), and visual impairment (*P* = 0.016) significantly decreased in patients requiring surgical treatment during the past decade (Fig. 2). Nevertheless, among complicated IVH patients who underwent surgical treatment (*n* = 404), a significantly higher incidence of HMD, BPD, PDA ligation, sepsis, NEC, PVL, and ROP was observed when compared with uncomplicated IVH patients who did not receive surgical treatment. Furthermore, patients who underwent surgery exhibited worse long-term neurological outcomes, including DD, CP, and seizure disorder, compared with the non-surgical group (*P* < 0.001) (Supplementary Table 1).

**Fig. 3:**
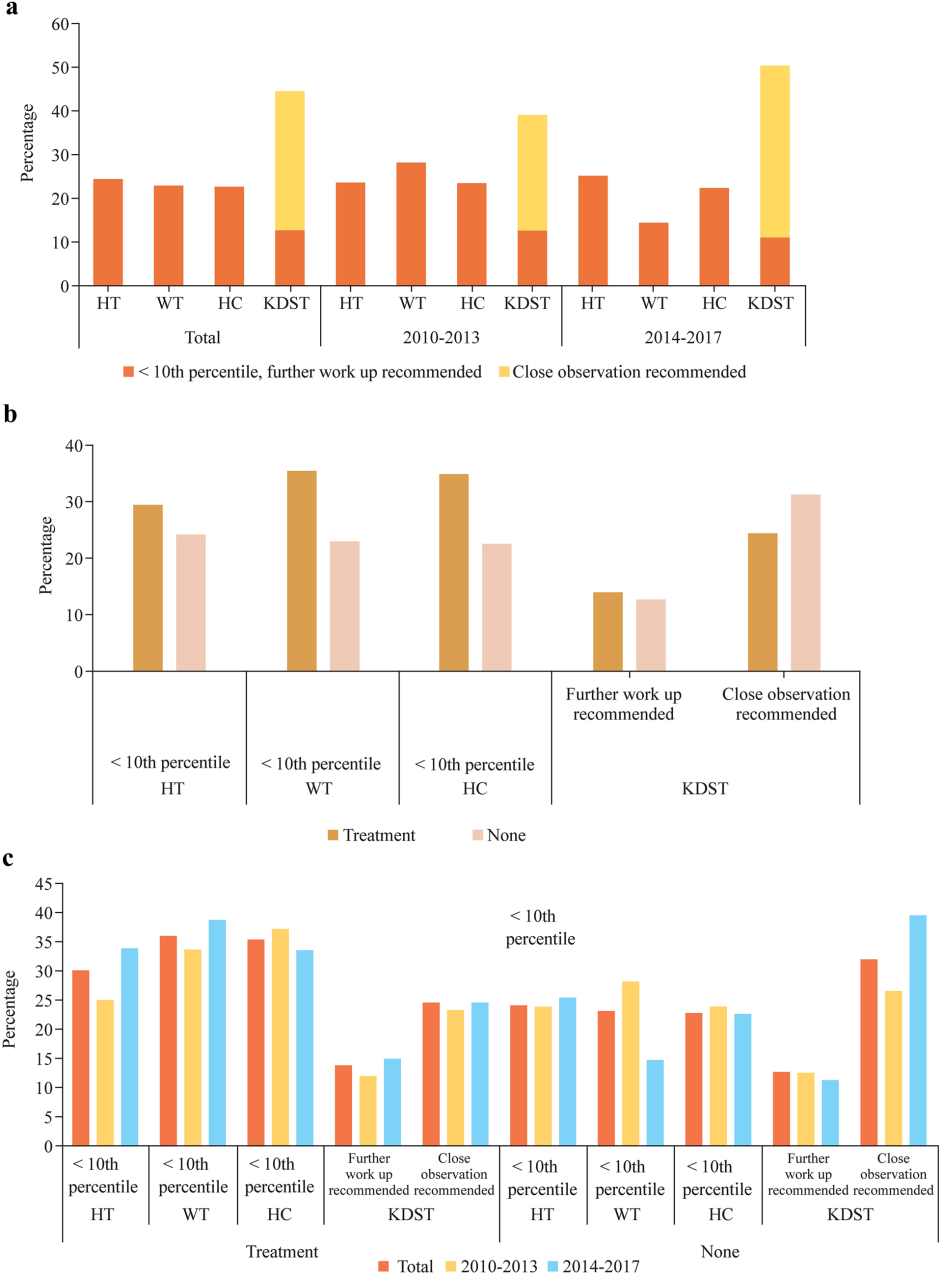
Incidence of infants with growth less than the 10th percentile and abnormal developmental outcome among VLBW infants with IVH. **a** Total period and division of the 8-year period into two halves of four years each; **b** dividing patients into two groups, with and without surgical treatment; **c** combination of group divisions by surgical treatment and the 8-year period into two halves of four years each. *P < 0.05. *IVH* intraventricular hemorrhage, *VLBW* very low birth weight, *HT* height, *WT* weight, *HC* head circumference, Korean Developmental Screening Test for Infants and Children (KDST)

Figure 3 illustrates the percentage of children with IVH who experienced growth below the 10th percentile and abnormal developmental outcomes as assessed by the K-DST. A total of 24% of infants exhibited height (HT) below the 10th percentile, 23% had weight (WT) below the 10th percentile, and 23% had a head circumference (HC) below the 10th percentile. Additionally, 13% of infants required further work-up, while 31% required close observation. When dividing study periods into two groups based on a period of years, 2010–2013 (358 infants) and 2014–2017 (329 infants), a significant decrease in the number of infants with weight below the 10th percentile (28% to 15%, *P* < 0.001) and requiring further work-up (13% to 11%, *P* < 0.05) was observed (Fig. 3a). Among the 404 infants with IVH who underwent surgical treatment, a significantly higher incidence of growth below the 10th percentile was observed, including HT (31% vs 24%, *P* < 0.001), WT (36% vs 23%, *P* < 0.001), and HC (36% vs 21%, *P* < 0.001). These infants also exhibited a higher occurrence of abnormal developmental outcomes (13% vs 11%, *P* < 0.05) (Fig. 3b). When dividing the years into two halves and by surgical treatment, IVH patients who underwent surgical therapy during the earlier 4 years exhibited poorer growth and more, requiring further work-up (*P* < 0.001) (Fig. 3c).

## Discussion

We observed a consistent rise in IVH incidence over the past decade, despite a decrease in mortality among VLBW infants. Notably, both short-term outcomes (such as BPD and ROP) and long-term outcomes (including CP and seizure disorder) have shown improvement over this period in VLBW infants with IVH. However, among infants with IVH who underwent surgical treatment (10%), there was a tendency towards more severe co-morbidities and long-term sequelae, such as CP, DD, and seizure disorder. Additionally, these infants exhibited a higher prevalence of growth below the 10th percentile in HT, WT, and HC, as well as poorer developmental test results, compared with those without treatment. It is noteworthy that significant improvements were observed when dividing the study period into two halves of 4 years each. This study, encompassing almost all infants, as per information from the Korean Statistical Information Service, provides valuable insights into the nationwide epidemiology of IVH over the past decade.

Advancements in neonatal intensive care have led to improved survival rates for extremely preterm infants [23]. Stoll et al. argued that the effort to save more high-risk infants has resulted in an increased proportion of IVH. They reported that the decreasing trend of IVH rates, which reached 14% in 2002, either stabilized or showed a relative increase, reaching 16% in 2007 [5, 6]. The global incidence range of IVH G3 and G4 was reported to be 6%–22%, with variations by region (Europe: 6%–17%; North America: 11%–22%; Asia: 10%–14%; Oceania: 12%–13%) [14]. IVH G2 had an incidence range of 5%–19%. Among infants with a gestational age of less than 24 weeks, the incidence of IVH was close to 37%. Generally, the incidence of IVH was inversely related to gestational age. In our cohort, the overall prevalence of IVH (≥ G2) in VLBW infants was 12.1%. It is worth noting that Stoll et al. observed increasing trends in both BPD and IVH, whereas our cohort exhibited a decreasing trend in BPD [5]. This disparity may be attributed to the fact that their measurements were taken from a slightly earlier era, during which ventilator strategies for BPD and post-natal corticosteroid administration have evolved over the past decade [24, 25].

IVH is closely linked to increased mortality and neurodevelopmental abnormalities. Notably, neonates with IVH G3 and G4 are at high risk of experiencing long-term neurologic and neurodevelopmental disabilities, including seizures, cognitive and executive function impairment, and CP [2–4]. In Korea, the mortality rate among VLBW infants stood at approximately 20%, with IVH-related mortality accounting for 7% of this total. Subsequent follow-up studies, conducted until the age of 22, unveiled those individuals with IVH faced a 4.2-fold increased risk of developing CP [11]. Furthermore, the risk of cardiovascular, respiratory, digestive, eye and ear issues, as well as malignant tumors, continued to rise, leading to a lifelong burden for patients [26–28]. IVH patients exhibited a 2.68-fold increased risk of having an intelligence quotient (IQ) below 2 SD, a 4.45-fold increased risk of motor dysfunction, and a 2.91-fold increased risk of encountering difficulties in any academic skills (below 2 SD) [29–31]. In this study, CP was diagnosed in 837 patients with IVH (28%), and DD was observed in 1095 patients (36%). While it is about known that CP occurs in about 20–30% of cases after IVH, long-term outcomes following ventricular drainage have not been extensively studied [32]. The data from a single-center cohort revealed that the mortality rate of IVH was 36.9%. Among the 77 surviving patients, the rate of surgical treatment after PHVD was 18%, and the incidence of CP was 55.9% (49.4%) [33].

In some cases, infants who are unable to tolerate a permanent shunt due to specific conditions may undergo a temporizing surgical procedure to divert cerebrospinal fluid until these issues are resolved. Published rates of conversion from a temporary device to a permanent shunt in this population vary, ranging from just over 50% to as high as 85% [16, 17, 34–36]. In 2019, the Early Versus Late Intervention Study (ELVIS) Trial group in the Netherlands demonstrated the highest recorded conversion rate, at 26% [37, 38]. In this study, temporary devices were used in 1.2% of IVH VLBW infants, while permanent shunts were required in 4.8% of cases. The conversion rate from temporary to permanent devices was 42.3%.

Notably, the risk of a poor neurodevelopmental outcome is significantly higher when severe IVH is complicated by PHVD, with a prevalence ranging from 40 to 60%. Further risk is observed in infants who eventually require a shunt, with prevalence as high as 75% to 88% [14, 15]. In the pre-surfactant era, up to 82% of infants with PHVD who survived developed significant neurologic impairments, including CP [39]. In our study, CP was reported in 245 patients (76%) in the surgical intervention group, which was higher than the 77 patients (24%) in the non-surgical intervention group.

In our study, the K-DST identified 16 patients (12.8%) categorized as requiring “further work-up”. When dividing the study period into the first half and the more recent half, the percentage was 12.7% from 2010 to 2013 and 11.3% from 2014 to 2017. These findings suggest that improved neonatal care and high-risk infant follow-up care have led to a significant reduction in abnormal neurodevelopmental results. However, among patients who underwent surgical treatment, the percentage of those categorized as “further work-up” was 13%, compared with 11% of infants without treatment. This highlights the need for careful consideration and early intervention in such cases.

This study boasts several strengths, notably its nationwide scope, enabling a comprehensive epidemiological analysis of IVH and PHVD in VLBW infants across all live births. Additionally, the analysis included long-term growth and developmental screening data until the age of six years.

However, it is crucial to acknowledge certain limitations in this study. Due to the nature of national health claim data, access to individual patient data was not possible. The study design was observational, and patient inclusion relied solely on ICD-10 codes, which may have introduced labeling errors or missed diagnoses from various hospitals that could not be rectified. The surgically treated group can be considered as having PHVD; however, it is important to note that treatment approaches vary among different institutes, introducing an additional challenge in drawing conclusive findings. We can only make assumptions that patients who receive surgical treatment might represent a more complex spectrum of the disease. Controlling for the heterogeneity and subjectivity in measuring scales across various units and clinicians was not feasible in this retrospective observational study. Furthermore, recent studies have proposed distinct outcomes associated with periventricular hemorrhagic infarction, suggesting the implication of different etiologies, as described by Volpe et al., rather than the traditional description by Papile et al. [4, 39, 40]. The diagnosis was applied according to the ICD-10 code inputted by the hospital. It was not possible to distinguish which diagnostic criteria were used between the Papile and Volpe criteria. To further enhance our understanding, future data collection efforts should categorize cases according to the provided diagnostic classification. While the K-DST was used as a developmental screening tool, the Bayley Scales of Infant Development were not employed as a diagnostic tool. Additionally, the study did not assess the association between outborn birth status and mortality or morbidity. Lastly, the duration of follow-up data was relatively short, potentially leading to an underestimation of developmental disorders that typically manifest after the age of three.

In conclusion, despite advancements in current neonatal care, the neurodevelopmental outcomes of infants with IVH, especially those who undergo surgical treatment, continue to be a matter of concern. It is imperative to prioritize specialized care for patients receiving surgical treatment and closely monitor their growth and development after discharge to improve developmental prognosis.

### Supplementary Information

Below is the link to the electronic supplementary material.

Supplementary file1 (DOCX 19 kb)

## Data Availability

The dataset analyzed in this study is not publicly available due to the policy of Research of Korea Centers for Disease Control and Prevention. However, the dataset is available on reasonable request.
