# Supplementary material for: Long-term outcomes of very low birth weight infants with intraventricular hemorrhage: a nationwide population study from 2011 to 2019
Source: World J Pediatr. 2024 Apr 13;20(7):692–700. doi: 10.1007/s12519-024-00799-x (PMC11269332; doi:10.1007/s12519-024-00799-x)
Supplement: Supplementary file 2 — Supplementary file1 (DOCX 19 kb) [file 12519_2024_799_MOESM1_ESM.docx]

Supplementary Table 1 Comparisons of co-morbidities and long-term outcomes in IVH Infants with and without surgical treatment

| Variables | IVH with surgical treatment (*n* = 404) | IVH without surgical treatment (*n* = 3401) | *P*-value |
| --- | --- | --- | --- |
| HMD | 391 (97%) | 2929 (86%) | < 0.001 |
| BPD | 345 (85%) | 2145 (63%) | < 0.001 |
| PDA ligation | 140 (35%) | 656 (19%) | < 0.001 |
| Sepsis | 209 (52%) | 1397 (41%) | < 0.001 |
| NEC | 99 (25%) | 522 (15%) | < 0.001 |
| PVL | 84 (21%) | 492 (15%) | 0.001 |
| ROP | 262 (65%) | 1966 (58%) | 0.007 |
| Mortality | 62 (15%) | 556 (16%) | 0.606 |
| Delayed development | 236 (58%) | 1058 (32%) | < 0.001 |
| Cerebral palsy | 289 (72%) | 676 (20%) | < 0.001 |
| Autism spectrum disorders | 23 (6%) | 142 (4%) | 0.157 |
| Sensorineural hearing loss | 42 (10%) | 327 (10%) | 0.616 |
| Blindness | 6 (2%) | 23 (1%) | 0.077 |
| Seizure disorders | 314 (78%) | 768 (23%) | < 0.001 |

*HMD* hyaline membrane disease, *BPD* bronchopulmonary dysplasia, *PDA* patent ductus arteriosus, *NEC* necrotizing enterocolitis, *PVL* periventricular leukomalacia, *ROP* retinopathy of prematurity
